# Lateralization of increased density of Iba1-immunopositive microglial cells in the anterior midcingulate cortex of schizophrenia and bipolar disorder

**DOI:** 10.1007/s00406-020-01107-0

**Published:** 2020-02-15

**Authors:** Elisabeth Petrasch-Parwez, Andreas Schöbel, Alia Benali, Zahra Moinfar, Eckart Förster, Martin Brüne, Georg Juckel

**Affiliations:** 1 Department of Neuroanatomy and Molecular Brain Research, Institute of Anatomy, Ruhr-University of Bochum, Bochum, Germany; 2 Section for Computational Sensomotorics, Department of Cognitive Neurology, Hertie-Institute for Clinical Brain Research and Centre for Integrative Neuroscience, University of Tübingen, Tübingen, Germany; 3 International Graduate School of Neuroscience, Ruhr-University of Bochum, Bochum, Germany; 4 Division of Social Neuropsychiatry and Evolutionary Medicine, Department of Psychiatry, LWL University Hospital Bochum, Bochum, Germany; 5 Department of Psychiatry, LWL University Hospital Bochum, Ruhr-University of Bochum, Bochum, Germany; 6 Klinik für Psychiatrie, Psychotherapie und Präventivmedizin, LWL- Universitätsklinikum der Ruhr Universität Bochum, Alexandrinenstr.1, 44791 Bochum, Germany

**Keywords:** Microglia, Schizophrenia, Bipolar disorder, Anterior midcingulate cortex (aMCC), Lateralization, Ionized calcium binding adaptor molecule 1 (Iba1)

## Abstract

There is increasing evidence from genetic, biochemical, pharmacological, neuroimaging and post-mortem studies that immunological dysregulation plays a crucial role in the pathogenesis of psychoses. The involvement of microglia in schizophrenia and bipolar disorder (BD) has remained controversial, however, since results from various post-mortem studies are still inconclusive. Here, we analyzed the estimated density of microglia of age-matched individuals with schizophrenia (*n* = 17), BD (*n* = 13), and non-psychiatric control subjects (*n* = 17) in the anterior midcingulate cortex (aMCC), a brain area putatively involved in the pathogenesis of psychoses, using ionized calcium binding adaptor molecule 1 (Iba1)—immunohistochemistry. The microglial cells displayed a homogenously distributed Iba1—staining pattern in the aMCC with slightly varying activation states in all three groups. The estimated microglial densities did not differ significantly between individuals with schizophrenia, BD and control subjects. Remarkably, when both hemispheres were investigated separately within the three groups, the density was significantly lateralized towards the right aMCC in schizophrenia (*p* = 0.01) and—even more evident—in BD subjects (*p* = 0.008). This left–right lateralization was not observed in the control group (*p* = 0.52). Of note, microglial density was significantly lower in BD individuals who did not commit suicide compared with BD individuals who died from suicide (*p* = 0.002). This difference was not observed between individuals with BD who committed suicide and controls. The results, tentatively interpreted, suggest a hitherto unknown increased lateralization of microglial density to the right hemisphere in both psychiatric groups. If confirmed in independent samples, lateralization should be considered in all post-mortem studies on microglia. Density differences between suicide and non-suicide individuals needs further elucidation.

## Introduction

Microglia cells are among the key players in the central neuro-immune system. They survey the regional environment of the brain by rapid response to any physiological and/or pathological change. There is increasing evidence from genetic, biochemical, pharmacological, neuroimaging and post-mortem studies that immunological dysregulation plays a crucial role in the pathogenesis of psychiatric disorders such as schizophrenia and bipolar disorder (BD; reviewed by [1, 2]). Changes in the morphology and density of microglia have been described in various studies, the majority of which has been performed in brain tissue derived from post-mortem tissue from deceased individuals with schizophrenia and, to a lesser extent, BD. These studies have shown similarities and differences in the innate immune response in these two disorders [3].

In schizophrenia, activated microglia may correspond either to a reaction of the immune system to a continuous dysregulation of processes involved in neuroplasticity [4] or to an early answer to any (non-) inflammatory event during pregnancy, potentially followed by microgliosis due to an elevated maternal cytokine level [5]. The latter hypothesis is supported by animal studies [6, 7].

A meta-analysis on post-mortem studies of microglia in schizophrenia indicated an increase in microglial density in various brain areas with considerable heterogeneity between the investigations [8]. For instance, Bayer et al. [9] performed a qualitative post-mortem study using a human leucocyte antigen (HLA)—DR antibody, and detected activated microglia in the frontal cortex and hippocampus of three individuals with late-onset schizophrenia. Radewicz et al. [10] found an increased density of HLA-DR-positive microglial cells in older patients with chronic schizophrenia in the dorsolateral prefrontal and superior temporal cortex, but only a trend toward microgliosis in the anterior cingulate cortex (ACC). Wierzba-Bobrowicz et al. [11] reported on elevated density of HLA-DR-immunostained microglia in the frontal and temporal cortex of middle-aged schizophrenic female individuals. In contrast, Steiner et al. [12] detected no differences in HLA-DR-labeled microglial density in various brain areas including the ACC, but detected a highly elevated density in suicide victims with schizophrenia, also confirmed in a subsequent study in subjects with major depression and BD [13]. More recently, Fillman et al. [14] detected an increase in the number of microglia in the dorsolateral prefrontal cortex in individuals with schizophrenia. The studies mentioned above focused on microglial abnormalities using an HLA-DR antibody, which is generally considered as a marker mainly for activated microglia. In contrast, Hercher et al. [15] and Schnieder et al. [16] examined the microglial density in the white matter of several brain areas using an ionized calcium binding adaptor molecule (Iba1) antibody, an established marker which shows specific immunostaining of all resting and activated microglial cells in human brain tissue [17]. Hercher et al. [15] did not detect any difference between schizophrenia, BD and control individuals concerning microglial density. Schnieder et al. [16] found an increase in microglial density in the ventral half of the frontal cortex at the level of the genu of the corpus callosum in suicide subjects, not observed in non-suicide individuals, suggesting that altered immune regulation may also affect complex behaviors, including suicide. Taken together, the current data on microglia in patients with schizophrenia are inconsistent, partly contradictory and many questions concerning the role of microglia for schizophrenic pathophysiology remain unexplained.

BD is also a severe psychiatric illness with inflammatory and immunological processes also discussed contributing to the pathophysiology of this disorder as recently reviewed by Gridharan et al. [18]. Thus, an altered cytokine level during symptomatic and asymptomatic periods of BD was documented [19]. Rao et al. [20] found a significant upregulation of microglial markers in post-mortem frontal cortex in 10 BD patients. Steiner et al. [21] reported on an increased microglial density (immunostained with HLA-DR) in the pregenual ACC of seven BD patients. In contrast, a recent study showed that Iba1-immunostained microglia in various brain areas of BD patients is not immune activated [22].

Considering the so far controversial post-mortem results, we sought to investigate the estimated mean microglial density in the grey matter of the anterior midcingulate cortex (aMCC) of individuals who suffered from schizophrenia or BD, as well as from non-psychiatric control subjects by Iba1-immunohistochemistry. Since the aMCC, a separate functional and structural unit of the cingulate cortex [23] presumably constitutes a critical interface in schizophrenia and BD, tissue samples were chosen from this area for the investigation. As neuroinflammation appears to be linked to suicide [12] suicide subgroups were considered separately.

Decreased structural and functional hemispheric asymmetry is common in schizophrenic individuals as substantiated by imaging, psychological and also post-mortem studies [24]. Lateralization studies were mainly focussed on neuronal macro- and microstructure, investigations on glia considering side differences are still sparse [12]. To elucidate the role of lateralization in microglial density, left and right microglial densities were also compared.

## Subjects and methods

### Human brain tissue

Post-mortem coronal brain sections of 47 age-matched individuals were obtained from the Stanley Foundation Neuropathology Consortium (SFNC, Chevy Chase, USA). The Stanley Medical Research Institute confirmed that all donors gave written consent to examine the brains for research purposes. All brains were collected as described [25]. The clinical diagnosis was assessed according to the DSM-IV criteria. Detailed information about the Stanley Brain Collection can be obtained from the website of the institute (www.stanleyresearch.org.). The study was also approved by the Ethics Committee of the Medical Faculty of the Ruhr University Bochum, Germany (number 3165-08).

Seventeen subjects had the clinical diagnosis of schizophrenia, 13 of BD and 17 individuals served as controls, all of which with no known neurological or psychiatric disorder. The investigated demographic and clinical background data available from SFNC were documented in Table 1. In the schizophrenia group, sections of 10 individuals were taken from the left and seven from the right hemisphere. In the BD group eight were from the left and five from the right ACC, and the controls included 10 from the left and seven from the right side. Six of the 13 non-suicide schizophrenia individuals died from cardiac failure, two from overdose, two from pneumonia, one from pulmonary embolism, one from cirrhosis, and one from acute pancreatitis. Three of the eight non-suicide BD subjects died from cardiac failure, three from overdose, one from myocarditis and one from drowning. Finally, four of the schizophrenic (two hanging, two overdose) and five of the BD patients (three hanging, two overdose) had committed suicide. All 17 control patients died from cardiac failure.

**Table 1:** Demographic information, clinical background, brain-related data and estimated microglial density per mm^3^ in individuals with schizophrenia (Schiz), bipolar disorder (BD) and non-psychiatric controls

Variable	Schiz (*n* = 17)	BD (*n* = 13)	Controls (*n* = 17)	*F* value	*p* value
Age at death (yrs)	45.3 ± 6.7	46.0 ± 7.9	45.4 ± 5.7	0.127	0.88
Gender ratio (F:M)	06:11	09:04	04:13	0.044	0.84
Age at onset (yrs)	21.6 ± 6.5	24.1 ± 7.8		0.905	0.44
Duration of illness (yrs)	22.9 ± 11.2	21.9 ± 4.8		0.28	0.598
Brain weight (g)	1405 ± 111	1399 ± 135	1473 ± 154	4.493	0.013
Brain pH	6.49 ± 0.242	6.52 ± 0.295	6.69 ± 0.243	2.958	0.062
Post-mortem interval (h)	30.4 ± 11.9	35.2 ± 13.6	29.1 ± 13.8	2.550	0.082
Microglia/mm^3^	5529 ± 1100	5956 ± 927	6020 ± 1229	2.585	0.079

F, female; g, grams; h, hours; M, male; yrs, years

According to the information by the SFNC, the tissue was fixed in 10% phosphate-buffered formalin (No.: SF10020 Fisher Scientific, Waltham, MA, USA) for 8.5 weeks. Then brain slices were dissected in defined areas, transferred through graded phosphate-buffered sucrose solutions, frozen in 30% sucrose and cryostored. Coronal blocks with the corpus callosum and the overlying aMCC (beginning just behind the genu of the corpus callosum) were cut into series of 60 µm cryosections, each of which was collected in tubes with phosphate-buffered 30% sucrose and cryostored until used.

### Iba1 peroxidase immunohistochemistry and cresyl violet-staining

**Fig. 1:**
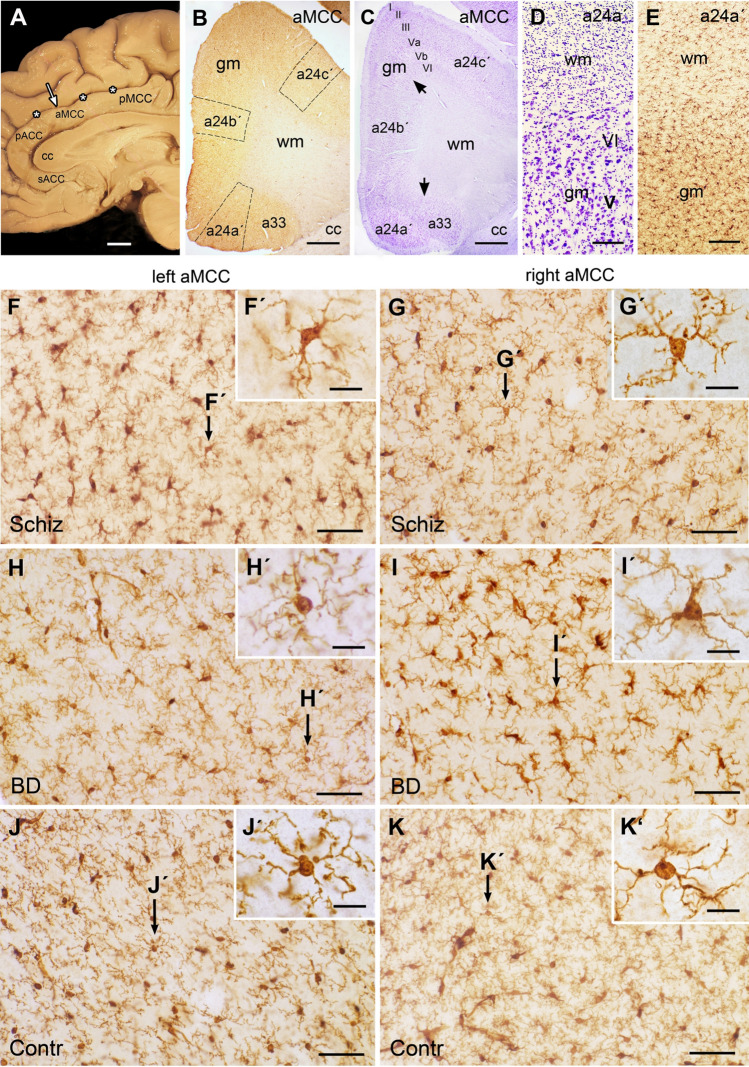
Investigated areas and morphology of microglia in the midcingulate cortex (aMCC). **a** Medial sagittal surface view shows the corpus callosum (cc), the overlying aMCC between the perigenual anterior cingulate (pACC) and posterior midcingulate cortex (pMCC) and the cingulate sulcus (asterisks). Arrow marks the investigated area. **b** Overview of the Iba1-stained cryosection shows the aMCC areas a24a′, a24b′ and a24c′ with the marked counted fields. **c** In the adjacent cresyl violet-stained section the borders between the subareas are marked by black arrows. Note the cortical layers I, II, III, Va, Vb, VI in the grey matter (gm). **d** Enlargement of the cresyl violet section shows the border area of white matter (wm) with abundant glial cells in contrast to the neurons in the cortical layers V and VI of the gm. **e** Note the difference in Iba1-staining between wm and gm. In the wm the distribution of microglial cell processes are oriented between the light appearing myelinated nerve fibers; in the gm numerous branching microglial processes extend in all directions from the somata. **f**–**k** Micrographs of Iba1-stained examples show the left (**f**, **h**, **j**) and right aMCC (**g**, **i**, **k**) of schizophrenia (Schiz; **f**, **g**), bipolar disorder (BD; **h**, **i**) and controls (contr; **j**, **k**) all of which with ramified microglia with slightly varying phenotypes confirmed by the inserts (**f, g**′–**k**′). Bar in **a **= 1 cm; bar in **b** and **c **= 1 mm; bar in **d** and **e** = 100 µm, bar in **f**–**k **= 50 µm, bar in **f**′–**k**′ = 10 µm. *sACC* subcallosal anterior cingulate cortex

All sections investigated here were taken from the aMCC which is bordered rostrally by the perigenual ACC and caudally by the posterior midcingulate cortex (Fig. 1a). Of note, the aMCC is often named dorsal ACC, which is incorrect as the MCC with its anterior and posterior part is an own cingulate cortical area [26].

For immunohistochemistry, sections were rinsed in phosphate buffered saline (PBS) and subjected to the commercially available rabbit anti Iba1 antibody (Code No. 019-19741; Wako Pure Chemical Industries, Osaka, Japan) according to an immunoperoxidase protocol as previously described [27]. Briefly, the sections were reduced in 1% NaBH_4_ and incubated in a free-floating fashion for 30 min in PBS with 10% normal goat serum plus 0.3% Triton X-100, followed by an incubation with the Iba1 antibody diluted 1:2000 for 72 h at 4 °C in the same solution. Next sections were rinsed in PBS, preincubated with 0.1% bovine serum albumin in PBS for 1 h and incubated for 24 h at 4 °C with a biotinylated goat anti-rabbit secondary antibody (Vector Laboratories, Burlingame, CA 94010, USA). After blocking in PBS-Albumin at RT, sections were incubated for 4 h with the avidin-biotinylated peroxidase complex (Vector Laboratories) at RT. Peroxidase activity was visualized with 3,3′-diaminobenzidine (DAB). The reaction time was first controlled microscopically and then standardized for all sections for 10 min. Finally, sections were mounted on Superfrost Plus slides (Thermoscientific, Menzel, Braunschweig, Germany), air-dried for 1.5 h, dehydrated and coverslipped with Entellan new (Merck Chemicals, Darmstadt, Germany).

The polyclonal Iba1 antibody used was raised against a synthetic peptide corresponding to the C-terminus of Iba1, a calcium binding protein specifically localized in microglia and macrophages, but not detectable in neurons, astrocytes and oligodendrocytes [17]. Therefore, the antibody is an effective microglia/macrophages marker in human brain tissue [27]. All sections with incomplete or insufficient immunostaining were excluded from the study. To avoid any mistake in the recognition of the microglial somata, counterstaining was omitted. Archived cresyl violet-stained coronal section series of the aMCC adjacent to the Iba1-immunostained cryosections were also obtained from the SFNC. These sections were taken as morphological reference for the identification of cortex (cortical grey matter) and medulla (cortical white matter). Photo documentation was performed using an Olympus Microscope BH-2 equipped with an Olympus DP-71 camera (Olympus Optical, Japan) and the computer-assisted software analysis Cell A (Soft imaging system GmbH, Münster, Germany). All data were exported as TIFF files into Adobe Photoshop CS5 (Adobe Imaging Systems Inc., USA) for documentation. Images used in the same figure were adapted for brightness and contrast.

### Cell counting

Quantification of microglial cells was performed using the above mentioned Olympus Microscope BH-2, the Olympus DP-71 camera and the software analysis Cell A. For microglial counting three fields were defined for each Iba1-stained section as region of interest (ROI) localized in the areas a24a′, a24b′ and a24c′ (Fig. 1b). Counting was performed under the microscope by focussing through the section level. Each counted field comprised the whole cortical thickness with the layers I–VI. The cortical layers were identified in the Iba1-stained sections by the very adjacent cresyl violet section (Fig. 1c). The border between the cortical grey and white matter was identified by a drop of cellular density in the cresyl violet-stained enlarged section (Fig. 1d). The grey matter contains numerous Nissl-stained neurons, the white matter is characterized by abundant small glial cells. The microglial density and morphology also strikingly changed in the Iba1-immunostained adjacent sections at the border between the cortical layer VI and the white matter (Fig. 1e). In the white matter the slightly undulated microglial cell processes were oriented along, across or around the course of myelinated nerve fibers, whereas in the grey matter numerous microglial processes extend and branch in all directions (Fig. 1f–k; f′–k′).

Throughout the analyzed ROI, only clearly Iba1-immunostained microglial cells with distinct somata were counted. Immunopositive monocytes, which were localized in blood vessels, were excluded. The ROI for microglial counting was measured at 20 × and cell counting was performed at 400 × magnification in the whole delineated area. Cell counting and quantitative analyses were performed by two independent raters, both were blind to the diagnosis when counting. To control the concordance, sections of 10 subjects were counted by both raters. The mean concordance between the raters was 96.1% (range 93.4–100%).

As sections tend to shrink during the immunohistochemical procedure, shrinkage was determined by measuring each section in different areas with a calibrated microcope stage (Leica DMRB) as previously described [28, 29]. The mean thickness of the sections of all groups was 23.5 ± 3.2 µm. Significant differences regarding the section thickness were not observed neither between nor within the groups.

The numerical density was estimated using the formula: Nv = *Q*/*v* (dis), where *Q* is the average number of microglial cells counted per dissector (ROI) and *v* (dis) is the volume of the dissector: *v* (dis) = *a* [ROI] × *h*, where ‘a’ is area of ROI and ‘*h*’ is the dissector height multiplied with the shrinkage factor. Nv of microglial cells was estimated as number of microglial cells in 1 mm^3^. The ROI area and its height were measured for each ROI with the Neurolucida system (MicroBrightfield).

### Statistical analyses

Normal distribution of the data was confirmed by the Kolmogorov–Smirnov and the Shapiro–Wilk test. All results are shown as group means [± standard error of the mean (SEMs)]. Between-group differences were examined using univariate analysis of variance (ANOVA), followed by post hoc Tukey test (*α* < 0.05) with a Bonferroni correction for multiple comparison (see Table 1). There was homogeneity of the error variances, as assessed by Levine’s test (*p* > 0.05). The statistical analyses were performed using the Statistical Package for the Social Sciences (SPSS Inc., Chicago, Illinois) for windows. *p* values < 0.05 were considered to be statistically significant.

## Results

### Morphology of Iba1-immunostained microglia in the aMCC

Iba1-immunostaining showed a distinct and specific labeling of microglia in the grey matter of the aMCC. The cells were evenly distributed throughout the cortical layers and could easily be distinguished from the background (Fig. 1f–k). The Iba1-immunoreactivity was detected in microglia, perivascular macrophages and in monocytes. According to the commonly accepted microglial phenotypes [28] by Iba1-immunostaining in human post-mortem brain tissue, cells displayed mainly a ramified, primed (both resting) and reactive phenotype (Fig. 1f′–k′), while amoeboid cells or microglial nodules were rarely observed. Differences in the activation state were detected, but were also observed in individuals of all three groups without any obvious attribution neither to one of the three groups nor to the left or right aMCC. The morphological integrity of the aMCC was controlled by very adjacent cresyl violet-stained sections (Fig. 1c, d) also obtained by the SFNC. Cresyl violet-staining allows an evaluation of the cytoarchitecture and gross histopathological alterations including shrinkage of neuronal somata, and of nuclear, cytoplasmic and proximal dendritic appearance. All 47 individuals investigated here showed no evidence of neurodegenerative changes nor inflammatory cell infiltration in any cortical layer of the aMCC.

Of note, each brain had earlier been macrosopically and microcopically screened by certified neuropathologists by the SFNC to rule out obvious neuropathologies [25].

### Density of microglia in schizophrenia, bipolar disorder and control individuals

The estimated mean density of microglia in the aMCC revealed no significant difference between the three groups (ANOVA, *F*2.585; *p* = 0.079). The mean density was lowest in the schizophrenia group and highest in control individuals (Table 1), but did not reach significance (*p* = 0.088). Comparing the density of microglia between controls and BD individuals (*p* = 0.79) and between BD and schizophrenic subjects (*p* = 0.09), no significant differences could be detected.

To control the effect of gender, which was unevenly distributed within the groups (Table 1), the microglial density of female and male subjects was investigated as a second independent factor (ANOVA, posthoc LSD, with a Bonferroni correction for multiple comparisons). Significant differences between the microglial densities of females and males were not determined (*F* = 0.07, *p* = 0.35), neither in the schizophrenia (*p* = 0.35) nor in the BD (*p* = 0.83) or in the control group (*p* = 0.53).

### Lateralization of microglial density in schizophrenia and bipolar disorder

**Fig. 2:**
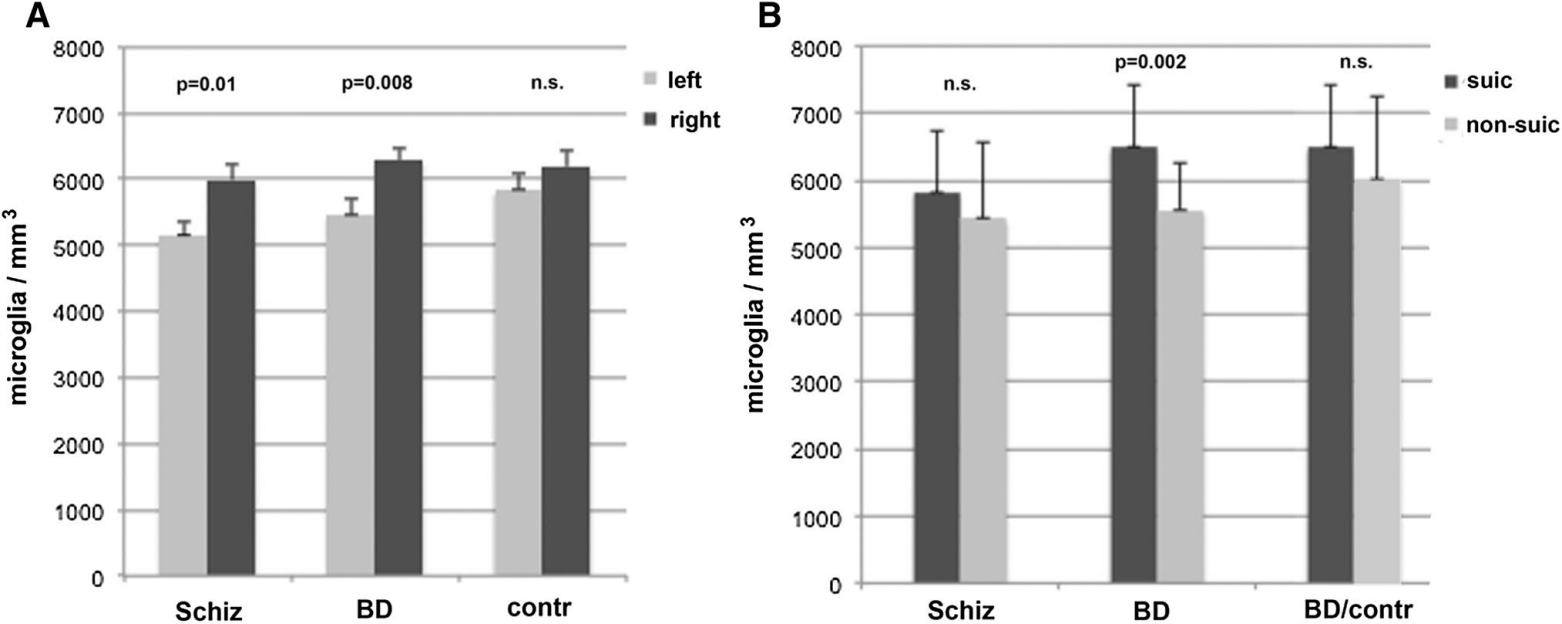
Comparison of microglial densities between the left and right anterior midcingulate cortex and between suicide (suic) and non-suicide (non-suic) individuals. **a** Significant difference in microglial density with a lateralization towards the right side is detected between the schizophrenia (Schiz) and the bipolar disorder BD individuals, not observed in the controls (contr). **b** Differences between non-suic and suic individuals are evident within the BD group; the BD non-suic subjects display a significantly lower microglial density compared to the suic victims. Comparing BD suic victims with controls, the difference is not significant. In the Schiz group, no significant difference emerges between suic and non-suic individuals regarding microglial density

As lateralization appears to play a major role in debates of psychotic disorders (see, e.g., Flor-Henry [30]), the density of each hemisphere was investigated between both aMCC of each group separately. The data revealed that there was no difference in density between the two hemispheres in the control group, whereas the difference within each patient group was remarkable (Fig. 2a). In schizophrenia, the difference between the left (5157.1 ± 205.7 MG/mm^3^) and the right side (5994.9 ± 230.1 MG/mm^3^) was clearly significant (*p* = 0.01). The lateralization to the right aMCC was even more pronounced in the BD individuals, who exhibited an estimated density of 6285.1 ± 184.4 MG/mm^3^ in the right aMCC in comparison to the left side with 5463.2 ± 225.8 MG/mm^3^ (*F* = 7.944, *p* = 0.008). Significant hemispheric differences were not observed within the control group (*p* = 0.52) showing a density of 6193.9 ± 245.6 MG/mm^3^ in the right and 5830.4 ± 256.1 MG/mm^3^ in the left aMCC.

### Microglial density in suicide victims

Four of the 17 schizophrenic and five of the 13 bipolar patients had committed suicide. When pooling the non-suicide (*n* = 21) versus the suicide victims (*n* = 9) of both psychiatric groups, the difference concerning microglial cells revealed a trend toward significance (*F* = 5.078, *p* = 0.08), when compared with control individuals who died from natural causes of death (Table 2). Surprisingly, the post hoc analysis showed that there was no significant difference in microglial cell density between the control group and the suicide individuals (*p* = 1). However, there was a difference between non-suicide patients and controls (*p* = 0.039) and between non-suicide and suicide patients (*p* = 0.018). The microglial cell density was decreased in the psychiatric non-suicide individuals compared to the other two groups.

**Table 2:** Comparison of demographic information, clinical background, brain-related data and estimated microglial density per mm^3^ in non-suicide (Schiz, *n* = 13; BD, *n* = 8) and suicide victims (Schiz, *n* = 4; BD, *n* = 5) of schizophrenia (Schiz) and bipolar disorder (BD) individuals compared to control subjects

Variable	Schiz + BD Non-suicide (*n* = 21)	Schiz + BD Suicide (*n* = 9)	Controls Non-suicide (*n* = 17)	*F* value	*p* value
Age at death (yrs)	46.5 ± 7.2	42.4 ± 7.7	45.4 ± 5.7	0.127	0.881
Sex ratio (F:M)	10:11	05:04	04:13	0.468	0.496
Age at onset (yrs)	20.8 ± 7.0	27.1 ± 5.8		17.784	0.000
Duration of illness (yrs)	25.7 ± 7.1	15 ± 9.3		37.831	0.000
Brain weight (g)	1375 ± 126.3	1466 ± 91.5	1471 ± 154	9.355	0.000
Post mortem interval (h)	32.7 ± 13.12	31.8 ± 12.7	29.1 ± 14.3	1.079	0.343
Microglial density/mm^3^	5474 ± 1004	6193 ± 970	6020 ± 1229	9.386	0.003

F, female, g, grams, h, hours; M, male; yrs, years

The suicide cases were also significantly older at the first onset of the disease, when compared to the non-suicide individuals (Table 2). Accordingly, the duration of illness was significantly shorter.

The suicide and non-suicide patients were also investigated separately within the patient groups (*F* = 9.386, *p* = 0.003). In schizophrenia, the microglial density of non-suicide patients (5424.48 ± 1157.24 MG/mm^3^) was decreased, but did not differ significantly (*F* = 1.129, *p* = 0.29) from the density of the schizophrenic suicide cases (5818.22 ± 909.56 MG/mm^3^). In contrast, in BD subjects the differences in density of microglia reached significance (*F* = 11.467, *p* = 0.002), when non-suicide (5554.40 ± 701.54 MG/mm^3)^ and suicide subjects (6492.38 ± 939.28 MG/mm^3^) were compared. Notable, when comparing the BD suicide group with the controls (6020 ± 1229 MG/mm^3^), the difference was not significant (Fig. 2b).

Age (*F* = 0.127, *p* = 0.881), post-mortem interval (*F* = 2.550, *p* = 0.082) and brain pH (*F* = 2.958, *p* = 0.062) had no significant effect on the microglia density (Table 1). The significant difference between the suicide and non-suicide victims regarding the age at onset and duration of illness turned out to be restricted to the schizophrenic group, the difference was not observed between suicide and non-suicide BD individuals.

## Discussion

In the present study we investigated the mean estimated density of Iba1-immunostained microglial cells in the aMCC of schizophrenia, BD and non-psychiatric control brains. The density of microglia was decreased in schizophrenia, but did not differ significantly from the density in BD and control patients. Of note, a metaanalysis on multiple schizophrenia studies most of which conducted with the CD68- and HLA-DR-antibody indicated an increase in microglial densities in many brain areas, but also showed remarkable heterogeneity between the studies [8]. Trépanier et al. [31] already reviewed that microglia marker could be increased, unchanged or even lowered in schizophrenia individuals. The variability across the investigations could be due to the source of the tissue, post-mortem interval, age at death, the area investigated and also to methodological reasons. It is likely that the application of different antibodies may also lead to varying results. The most widely used antibodies for staining microglia in human brain tissue are the HLA-DR and CD68 antibodies, only a few studies on human psychiatric brains used the Iba1 antibody. Immunostaining with these three microglia markers shows similar, but not identical staining pattern in human brain sections [32]. Therefore, the comparison of the microglial density between various studies is limited, when different antibodies are used. Taking into account which antibody had been used, our data are in line with corresponding studies by Hercher et al. [15], who investigated the dorsolateral prefrontal cortex by Iba1-immunohistochemistry of the three patient groups, also obtained from the SFNC. Schnieder et al. [16], who also used the Iba1 antibody, did not find significant microglial density differences between schizophrenia, BD and controls when investigating the white matter of the prefrontal cortex. Accordingly, in future studies various antibodies should be used to compare the different microglial staining pattern in the same brain.

Concerning BD, investigations on microglial densities are still sparse. Our results confirm the post-mortem results by Sneeboer et al. [22] showing that Iba1-stained microglial density in tissues from the BD patients did not differ between BD and control individuals. Sneeboer et al. [22] strengthened his results by a well designed multi-level study.

In our study, first the within-group comparisons between both hemispheres revealed a significant lateralization of microglial density towards the right side in both psychiatric groups, not observed in the controls. Lateralization plays a major role in debates on psychotic disorders (see, e.g., Flor-Henry [30]). There is an extensive literature to this topic, especially in schizophrenia [24], supporting the view of a reduced brain lateralization meaning a failed left hemisphere dominance as in healthy people. This could be caused by an altered connectivity among distinct cortical areas in such disorders. Concerning our results with higher microglial density on the right side, i.e., lower microglia cell density on the left side, it can be speculated whether the abolition of normal brain asymmetry has to due with the increase of this cell type on the right side (and depended cells and brain circuits) and followed compensation of the normal dysbalance in brain hemispheral structure and function. Our observation of a significant left–right lateralization of microglial density clearly indicates, that side differences should be taken into account in all microglia studies on human brain tissue. In a previous post-mortem study in schizophrenia, Steiner et al. [12] did not find side differences in the patients groups, but detected a significant lateralization of HLA-DR-stained amoeboid microglia towards the right hemisphere in healthy subjects. One reason for these contradictory results may again be the application of different antibodies. Furthermore, the difference observed by Steiner was restricted to the amoeboid microglia, whereas our studies included all microglial phenotypes. Due to the small sample size of ours and other post-mortem studies, these results should be interpreted with caution.

Application and duration of different fixatives could also influence the quality of immunostaining. Aqueous formaldehyde solutions often lead to poor immunoreactions when compared with immunostained tissue after buffered formaldehyde fixation. Fixed or unfixed frozen tissue may result in different or a lack of staining, cryo- and paraplast sections demand retrieval treatment prior to immunohistochemistry which may lead to various staining pattern. Finally, using either stereological or non-stereological counting also affect the results. Thus, the tissue treatment that precedes immunohistochemistry and the counting methods should also be carefully considered when comparing varying results.

It has previously been reported in various studies that suicide may be associated with microgliosis [12, 13, 16, 21]. In the present study, non-suicidal BD patients were characterized by a significantly lower microglial density in the aMCC compared to suicide BD individuals. Furthermore, when compared to controls, the decreased density of microglia in non-suicide individuals was confirmed. The difference in density is only in part in accordance with Steiner et al. [13], who first described a significantly higher number of microglial cells (stained by HLA-DR) of suicide patients with schizophrenia and depression in various brain areas including the perigenual ACC. Similar to our results, Brisch et al. [33] found that only tissue derived from the non-suicidal depressed subgroup revealed a significantly lower microglial reaction, i.e., a decreased density of HLA-DR positive microglia versus tissue from both depressed suicide victims and controls.

The Iba1-microglia staining showed a homogenous distribution throughout all cortical layers of the aMCC investigated in the patients as well as in the controls. This staining was in line with the Iba1 immunostaining of cortical areas documented by Schnieder et al. [16] in suicide and by Sneeboer et al. [22] in BD brains. Layer-specific differences in the density of HLA-DR-immunopositive microglia previously described by Wierzba-Bobrowicz et al. [11] between the outer and inner cortical layers of the ACC may be due to the different antibody used. In addition, the severe degeneration signs of numerous microglia observed by Wierzba-Bobrowicz et al. [34] in the cortex of patients suffering from chronic schizophrenia rarely occurred in the brain tissue investigated here in all groups including the control.

The present study has several limitations. First, our counting of microglial cells did not differentiate between the microglial phenotypes. The Iba1 antibody used here stains all microglia/macrophages independently from their activation state. Thus, it is still open what activity state the microglia may present in the post-mortem brains of patients with schizophrenia and BD individuals investigated here; qualitative inspection suggests that most microglial cells were of resting type in our three groups (Fig. 1). Moreover, conflicting results with respect to microglial density might also be due to the application of different microglial antibodies, each of which staining various and/or overlapping phenotypes of the heterogeneous population of microglia and macrophages within normal and diseased brains.

Finally, our sample size is small, especially in the suicide groups. Therefore, these results remain preliminary and should be interpreted with caution.

To conclude, in our study the density of microglia was significantly lateralized to the right aMCC in schizophrenic and BD individuals, which was not observed in controls. Furthermore, differences in microglial density were also observed between non-suicide and suicide BD victims showing a significant decrease of density in non-suicide individuals. The mechanism of these changes remains unknown. Future studies are needed to strengthen the hypothesis of lateralized microglial density in schizophrenia and BD by including a larger sample size of individuals, investigations on multiple brain regions and by the application of various antibodies.

## Abbreviations

ACC: Anterior cingulate cortex
aMCC: Anterior midcingulate cortex
BD: Bipolar disorder
Contr: Control individuals
Iba1: Ionized calcium binding adaptor molecule 1
F: Female
g: Grams
gm: (cortical) Grey matter
h: Hours
HLA: Human leucocyte antigen
M: Male
pMCC: Posterior midcingulate cortex
MCC: Midcingulate cortex
MG: Microglia cells
NGS: Normal goat serum
pACC: Perigenual anterior cingulate cortex
PBS: Phosphate-buffered saline
PFA: Paraformaldehyde
PMI: Post-mortem interval
ROI: Region of interest
sACC: Subcallosal anterior cingulate cortex
Schiz: Schizophrenia
SFNC: Stanley Foundation Neuropathology Consortium
SPSS: Statistical Package for the Social Sciences
suic: Suicide
wm: (cortical) White matter
yrs: Years
I: Molecular layer
II: External granular layer
III: External pyramidal layer
V: Internal pyramidal layer
VI: Multiform layer

## References

[1] Drexhage RC, Knijff EM, Padmos RC, Lv Heul-Nieuwenhuijzen, Beumer W, Versnel W, Drexhage HA (2010). The mononuclear phagocyte system and its cytokine inflammatory networks in schizophrenia and bipolar disorder. Expert Rev Neurother.

[2] Prinz M, Priller J (2014). Microglia and brain macrophages in the molecular age: from origin to neuropsychiatric disease. Nat Rev Neurosci.

[3] de Baumont A, Maschietto M, Lima L, Carraro DM, Olivieri EH, Fiorini A, Barreta LA, Palha JA, Belmonte-de-Abreu P, Moreira Filho CA, Brentani H (2015). Innate immune response is differentially dysregulated between bipolar disease and schizophrenia. Schizophr Res.

[4] Morara S, Colangelo AM, Provini L (2015). Microglia-induced maladaptive plasticity can be modulated by neuropeptides in vivo. Neural Plast.

[5] Rothermundt M, Arolt V, Peters M, Gutbrodt H, Fenker J, Kersting A, Kirchner H (2001). Inflammatory markers in major depression and melancholia. J Affect Disord.

[6] Juckel G, Manitz MP, Brüne M, Friebe A, Heneka MT, Wolf RJ (2011). Microglial activation in a neuroinflammational animal model of schizophrenia—a pilot study. Schizophr Res.

[7] Manitz MP, Esslinger M, Wachholz S, Plümper J, Friebe A, Juckel G, Wolf R (2013). The role of microglia during life span in neuropsychiatric disease—an animal study. Schizophr Res.

[8] van Kesteren CFMG, Gremmels H, de Witte LD, Hol EM, van Gol AR, Falkai PG, Kahn RS, Sommer IEC (2017). Immune involvement in the pathogenesis of schizophrenia: a meta-analysis on postmortem brain studies. Transl Psychiatry.

[9] Bayer TA, Buslei R, Havas L, Falkai P (1999). Evidence for activation of microglia in patients with psychiatric illnesses. Neurosci Lett.

[10] Radewicz K, Garey LJ, Gentleman SM, Reynolds R (2000). Increase in HLA-DR immunoreactive microglia in frontal and temporal cortex of chronic schizophrenics. J Neuropathol Exp Neurol.

[11] Wierzba-Bobrowicz T, Lewandowska E, Lechowicz W, Stepien T, Pasennik E (2005). Quantitative analysis of activated microglia, ramified and damage of processes in the frontal and temporal lobes of chronic schizophrenics. Folia Neuropathol.

[12] Steiner J, Mawrin C, Ziegler A, Bielau H, Ullrich O, Bernstein HG, Bogerts B (2006). Distrubution of HLA-DR-positive microglia in schizophrenia reflects impaired cerebral lateralization. Acta Neuropathol.

[13] Steiner J, Bielau H, Brisch R, Danos P, Ullrich O, Mawrin C, Bernstein HG, Bogerts B (2008). Immunological aspects in the neurobiology of suicide: elevated microglial density in schizophrenia and depression is associated with suicide. J Psychiatr Res.

[14] Fillman SG, Cloonan N, Catts VS, Miller LC, Wong J, McCrossin T, Cairns M, Weickert CS (2013). Increased inflammatory markers identified in the dorsolateral prefrontal cortex of individuals with schizophrenia. Mol Psychiatry.

[15] Hercher C, Chopra V, Beasley CL (2014). Evidence for morphological alterations in prefrontal white matter glia in schizophrenia and bipolar disorder. J Psychiatry Neurosci.

[16] Schnieder TP, Trencevska I, Rosoklija G, Stankov A, Mann JJ, Smiley J, Dwork AJ (2014). Microglia of prefrontal white matter in suicide. J Neuropathol Exp Neurol.

[17] Imai Y, Ibata I, Ito D, Ohsawa K, Kohsaka S (1996). A novel gene 1 in the major histocompatibility complex class III region encoding an EF hand protein expressed in a monocytic lineage. Biochem Biophys Res Commun.

[18] Gridharan VV, Sayana P, Pinjari OF, Ahmad N, da Rosa MI, Quevedo J, Barichello T (2019). Postmortem evidence of brain inflammatory markers in bipolar disorder: a systematic review. Mol Psychiatry.

[19] Barbosa IG, Morato IB, de Miranda AS, Bauer ME, Soares JC, Teixeira AL (2014). A preliminary report of increased plasma levels of IL-33 in bipolar disorder: further evidence of pro-inflammatory status. J Affect Disord.

[20] Rao JS, Harry GJ, Rapoport SI, Kim HW (2009). Increased excitotoxicity and neuroinflammatory markers in postmortem frontal cortex from bipolar disorder patients. Mol Psychiatry.

[21] Steiner J, Walter M, Gos T, Guillemin GJ, Bernstein HG, Sarnyai Z, Mawrin C, Brisch R, Bielau H, Meyer zu Schwabedissen L, Bogerts B, Myint AM (2011). Severe depression is associated with increased microglial quinolinic acid in subregions of the anterior cingulate gyrus: evidence for an immune-modulated glutamatergic neurotransmission?. J Neuroinflamm.

[22] Sneeboer MAM, Snijders GJLJ, Berdowski WM, Fernández-Andreu A, van Mierlo HC, Berdenis van Berlekom A, Litjens M, Kahn RS, Hol EM, de Witte LD, Psychiatric Donor Program of the Netherlands Brain Bank (NBB-Psy) (2019). Microglia in post-mortem brain tissue of patients with bipolar disorder are not immune activated. Transl Psychiatry.

[23] Vogt BA (2016). Midcingulate cortex: structure, connections, homologies, functions and diseases. J Chem Neuroanat.

[24] Ribolsi M, Koch G, Magni V, Di Lorenzo G, Rubino IA, Siracusano A, Centonze D (2009). Abnormal brain lateralization and connectivity in schizophrenia. Rev Neurosci.

[25] Torrey EF, Webster M, Knable M, Johnston N, Yolken RH (2000). The Stanley foundation brain collection and neuropathology consortium. Schizophr Res.

[26] Petrasch-Parwez E, Nguyen HP, Löbbecke-Schumacher M, Habbes HW, Wieczorek S, Riess O, Andres KH, Dermietzel R, von Hörsten S (2007). Cellular and subcellular localization of Huntingtin [corrected] aggregates in the brain of a rat transgenic for Huntington disease. J Comp Neurol.

[27] Torres-Platas SG, Cruceanu C, Chen GG, Turecki G, Mechawar N (2014). Evidence for increased microglial priming and macrophage recruitment in the dorsal anterior cingulate white matter of depressed suicides. Brain Behav Immun.

[28] Benali A, Leefken I, Eysel UT, Weiler E (2003). A computerized image analysis system for quantitative analysis of cells in histological brain sections. J Neurosci Methods.

[29] Benali A, Trippe J, Weiler E, Mix A, Petrasch-Parwez E, Girzalsky W, Eysel UT, Erdmann R, Funke K (2011). Theta-burst transcranial magnetic stimulation alters cortical inhibition. J Neurosci.

[30] Flor-Henry P (2010). Electrophysiological approaches to psychopathology and the influence of lateralization. Clin EEG Neurosci.

[31] Trépanier MO, Hopperton KE, Mizrahi R, Mechawar N, Bazinet RP (2016). Postmortem evidence of cerebral inflammation: a systematic review. Mol Psychiatry.

[32] Hendrixs DAE, van Eden CG, Schuurmann KG, Haman J, Huitinga I (2017). Staining of HLA-DR, Iba1 and CD68 in human microglia reveals partially overlapping expression depending on cellular morphology and pathology. J Immunol.

[33] Brisch R, Steiner J, Mawrin C, Krzyżanowska M, Jankowski Z, Gos T (2017). Microglia in the dorsal raphe nucleus plays a potential role in both suicide facilitation and prevention in affective disorders. Eur Arch Psychiatry Clin Neurosci.

[34] Wierzba-Bobrowicz T, Lewandowska E, Kosno-Kruszewska E, Lechowicz W, Pasennik E, Schmidt-Sidor B (2004). Degeneration of microglial cells in frontal and temporal lobes of chronic schizophrenics. Folia Neuropathol.

