# Correction to: Lateralization of increased density of Iba1-immunopositive microglial cells in the anterior midcingulate cortex of schizophrenia and bipolar disorder

**DOI:** 10.1007/s00406-021-01339-8

**Published:** 2021-10-12

**Authors:** Elisabeth Petrasch-Parwez, Andreas Schöbel, Alia Benali, Zahra Moinfar, Eckart Förster, Martin Brüne, Georg Juckel

**Affiliations:** 1 Department of Neuroanatomy and Molecular Brain Research, Institute of Anatomy, Ruhr-University of Bochum, Bochum, Germany; 2 Section for Computational Sensomotorics, Department of Cognitive Neurology, Hertie-Institute for Clinical Brain Research and Centre for Integrative Neuroscience, University of Tübingen, Tübingen, Germany; 3 International Graduate School of Neuroscience, Ruhr-University of Bochum, Bochum, Germany; 4 Division of Social Neuropsychiatry and Evolutionary Medicine, Department of Psychiatry, LWL University Hospital Bochum, Bochum, Germany; 5 Department of Psychiatry, LWL University Hospital Bochum, Ruhr-University of Bochum, Bochum, Germany; 6 Klinik für Psychiatrie, Psychotherapie und Präventivmedizin, LWL- Universitätsklinikum der Ruhr Universität Bochum, Alexandrinenstr.1, 44791 Bochum, Germany

## Correction to: European Archives of Psychiatry and Clinical Neuroscience (2020) 270:819–828 10.1007/s00406-020-01107-0

The article “Lateralization of increased density of Iba1-immunopositive microglial cells in the anterior midcingulate cortex of schizophrenia and bipolar disorder” written by Elisabeth Petrasch-Parwez, Andreas Schöbel, Alia Benali, · Zahra Moinfar, Eckart Förster, Martin Brüne and Georg Juckel was originally published electronically on the publisher’s internet portal on February 15, 2020 without open access. With the author(s)’ decision to opt for Open Choice the copyright of the article changed to © The Author(s) 2020 and the article is forthwith distributed under a Creative Commons Attribution 4.0 International License, which permits use, sharing, adaptation, distribution and reproduction in any medium or format, as long as you give appropriate credit to the original author(s) and the source, provide a link to the Creative Commons licence, and indicate if changes were made. The images or other third-party material in this article are included in the article’s Creative Commons licence, unless indicated otherwise in a credit line to the material. If material is not included in the article’s Creative Commons licence and your intended use is not permitted by statutory regulation or exceeds the permitted use, you will need to obtain permission directly from the copyright holder. To view a copy of this licence, visit http://creativecommons.org/licenses/by/4.0/. Open Access funding enabled and organized by Projekt DEAL.

The original article has been updated.

